# Electrochemical synthesis of mesoporous gold films toward mesospace-stimulated optical properties

**DOI:** 10.1038/ncomms7608

**Published:** 2015-03-23

**Authors:** Cuiling Li, Ömer Dag, Thang Duy Dao, Tadaaki Nagao, Yasuhiro Sakamoto, Tatsuo Kimura, Osamu Terasaki, Yusuke Yamauchi

**Affiliations:** 1 World Premier International (WPI) Research Center for Materials Nanoarchitectonics (MANA), National Institute for Materials Science (NIMS), 1-1 Namiki, Tsukuba, Ibaraki 305-0044, Japan; 2 Department of Chemistry, Bilkent University, 06800 Ankara, Turkey; 3 PRESTO and CREST, Japan Science and Technology Agency (JST), 4-1-8 Honcho, Kawaguchi, Saitama 332-0012, Japan; 4 Graduate School of Materials Science, Nara Institute of Science and Technology, 8916-5 Takayama, Ikoma, Nara 630-0192, Japan; 5 Department of Physics, Graduate School of Science, Osaka University, 1-1 Machikaneyama-cho, Toyonaka, Osaka 560-0043, Japan; 6 Advanced Manufacturing Research Institute, National Institute of Advanced Industrial Science and Technology (AIST), Shimoshidami, Moriyama, Nagoya 463-8560, Japan; 7 Graduate School of EEWS (BK21Plus), KAIST, Daejeon 305-701, Korea; 8 Department of Materials and Environmental Chemistry, EXSELENT, Stockholm University, 10691 Stockholm, Sweden; 9 Department of Nanoscience and Nanoengineering, Faculty of Science and Engineering, Waseda University, 3-4-1 Okubo, Shinjuku, Tokyo 169-8555, Japan

## Abstract

Mesoporous gold (Au) films with tunable pores are expected to provide fascinating optical properties stimulated by the mesospaces, but they have not been realized yet because of the difficulty of controlling the Au crystal growth. Here, we report a reliable soft-templating method to fabricate mesoporous Au films using stable micelles of diblock copolymers, with electrochemical deposition advantageous for precise control of Au crystal growth. Strong field enhancement takes place around the center of the uniform mesopores as well as on the walls between the pores, leading to the enhanced light scattering as well as surface-enhanced Raman scattering (SERS), which is understandable, for example, from Babinet principles applied for the reverse system of nanoparticle ensembles.

## Abstract (web summary)

The selective growth of mesoporous gold thin films with control over the porosity is a challenging task. Here, the authors report a soft-templating method allowing the growth of tunable, mesoporous gold films and examine their optical properties

## Graphical abstract

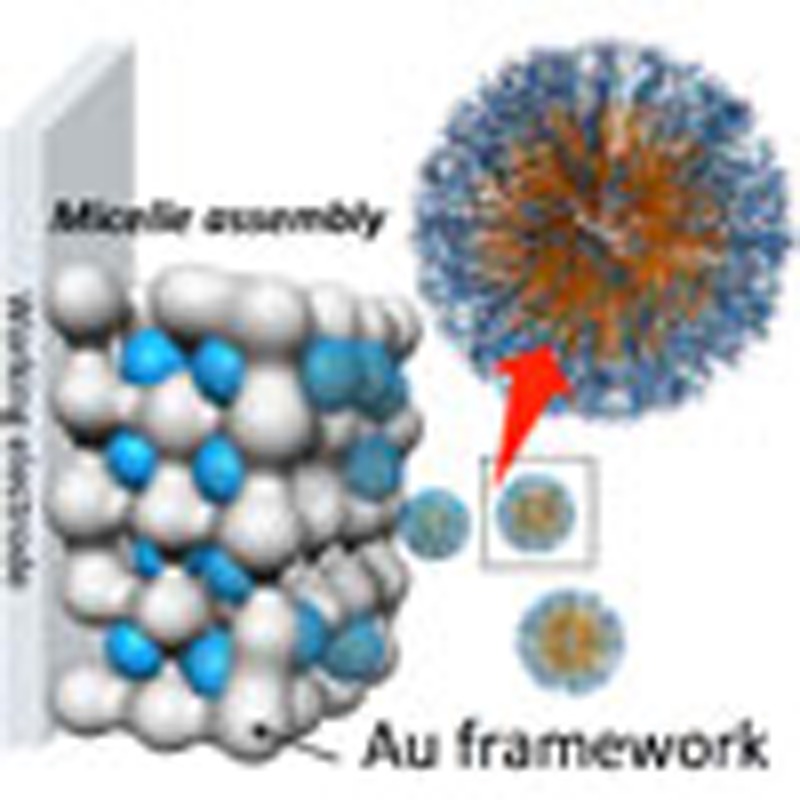

Nanostructural control is a quite important issue in materials chemistry to bring out unique chemical and physical properties. Mesoporous structures can steadily provide surfaces with many functional sites, which are critical for solving emergent problems. So far, many mesoporous materials with different compositions have been reported through self-assembly of amphiphilic organic molecules and keen interest has been shown because of their wide range of potential applications, including gas storage, separation, catalysis, ion-exchange, sensing, polymerization and drug delivery^1^,^2^,^3^. Especially, metallic mesoporous materials can exhibit rather high carrier density and thus remarkable optical response, which are not attainable by using other compositions of mesoporous materials (for example, silica-4,5 and carbon-based^6^,^7^ compositions). For example, free space radiation wavelength of the light in the near-infrared (NIR) to visible region are shrunk down by one to two orders of magnitude when they interact with the plasma oscillation of the free electrons at the metal surface^8^,^9^. The excited surface plasma oscillation, or surface plasmon, subsequently leads to the remarkable property that light can be manipulated flexibly by controlling the surface morphology of metals on the nanometer scale^10^,^11^,^12^,^13^. Considering working mechanisms of metal nanoparticles that have often been limited by their tendency to sinter to large-sized aggregates, three-dimensionally (3D) extended metal framework will steadily provide abundant reaction/adsorption sites, which are critical for designing emergently required functions.

Nanostructured gold (Au) materials in the forms of films and particles have not only shown high catalytic activities in several oxidation reactions (for example, CO, glucose, methanol) and O_2_ reduction reaction^14^,^15^,^16^,^17^,^18^ but also demonstrated unique optical properties due to their localized surface plasmon resonance (LSPR)^19^,^20^,^21^. Although various mesoporous metals and alloys have been prepared by soft- and hard-templating approaches^22^,^23^,^24^,^25^,^26^, Au thin films with uniform mesopores have not yet been obtained by such approaches, because of the difficulty in controlling the crystal growth of Au around the templates. In almost all the cases using hard-templates, nanostructured Au materials have only been obtained as nanoparticles, nanosheets and nanowires, without well-defined porous structures^27^,^28^. Although colloidal crystal templating is one of the most common techniques to form 3D ordered macroporous materials^29^,^30^,^31^,^32^, the use of small-sized particles is failed for templating of Au materials with periodic mesoporous structures (for example, inverse opal similar to 3D hexagonal structure)^33^. As far as we know, only one pathway to prepare mesoporous/nanoporous Au materials has been reported by dealloying treatment^34^,^35^,^36^, which is a selective dissolution process of less-noble metals in alloys^37^,^38^. However, this dealloying approach provides limited control over structural parameters and requires multistep processes under stringent experimental conditions, and nevertheless the resulting pores are irregular in size and shape. Thus, it is really difficult to shape Au crystals into mesoporous architectures.

A mesoporous Au material with tunable and uniform pores can newly be proposed as a reversed system of Au nanocrystals ensemble. Therefore, if we consider Babinet principle in optics, it is expected to show high-performance in light scattering as well as in surface-enhanced Raman scattering (SERS) for molecule detection due to multiple ‘hot spots’ built up in the mesopores as well as in the vicinity of narrow walls between the pores^39^.

Here, we report an effective way to prepare mesoporous Au films with fine tuning of the pore size by utilizing spherical micelles of polystyrene-*block*-poly(oxyethylene) (PS-*b*-PEO) diblock copolymers as soft-templates (Fig. 1a). In the electrolyte solution, HAuCl_4_ is dissolved into H_3_O^+^ and AuCl_4_^−^ ions and then interacts with the EO shells of the micelles through hydrogen bonding. This interaction favours H_3_O^+^ rather than AuCl_4_^−^, and consequently creates positively charged micelles that can be directed to the working electrode surfaces, where the AuCl_4_^−^ ions are, respectively, reduced to metallic Au with the electrochemical deposition of the micelles. The resultant mesoporous Au films actually exhibit high scattering performance and thus high activity for molecular sensing such as seen in SERS^40^ and surface-enhanced infrared absorption (SEIRA)^41^. Significantly, enhanced electric field (E-field) amplitude by excitation of LSPR is clearly seen inside or at the perimeter of the mesopores. The E-field amplitude and LSPR frequency are readily tunable by simply tuning the pore size and we could demonstrate the new methodology for tailoring the optical functionality.

## Results

### Fabrication of mesoporous Au films

**Figure 1:**
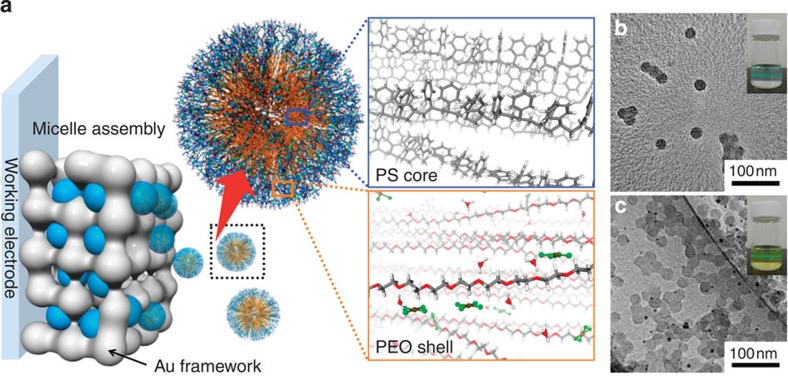
Synthetic concept of mesoporous Au films. (**a**) Schematic illustration of formation mechanism of mesoporous Au films through micelle assembly. (**b**,**c**) TEM images of PS_18000_-*b*-PEO_7500_ micelles formed in aqueous solution (with 3 ml THF) (**b**) without and (**c**) with HAuCl_4_ source. Black dots indicate Au nanoparticles formed on the surface of micelles by irradiation of electron beam. The Tyndall effect is also shown as an inset image.

Our approach is based on an electrochemical method, which has been practically utilized for preparation of continuous metallic films (Fig. 1a). For plating Au materials in the presence of PS-*b*-PEO micelles at the electrode surface, precursor solutions were prepared as follows. PS-*b*-PEO diblock copolymer was completely dissolved in tetrahydrofuran (THF), being a good solvent for the PS block, as its unimer. No micelles and/or aggregates were detected in the clear PS-*b*-PEO/THF solution by dynamic light scattering. The use of THF was necessary for the preparation; PS-*b*-PEO cannot be dissolved in water or ethanol without THF. After successive addition of ethanol, aqueous solution of HAuCl_4_ was slowly dropped to the solution and consequently spherical micelles of PS-*b*-PEO was formed on the basis of less solubility of the PS core in water. As shown in Fig. 1b,c, spherical micelles constructed by the PS cores and the PEO shells were observed by transmission electron microscopy (TEM) and their average diameter was ~25 nm. The Tyndall effect was also observed in the electrolyte (Fig. 1b,c). As illustrated in Fig. 1a, the hydrophilic EO blocks can interact with aqua-HAuCl_4_ complexes near the outer layer of the micelles, which will be discussed later. To directly confirm the presence of PS-*b*-PEO micelles in the electrolyte solution, we visualized uniformly sized polymeric micelles with the Brownian motion by a confocal laser scanning microscope. By applying potentials, the composite micelles that interacted with Au species were approached to deposit as mesostructured Au materials over the working electrode.

Block copolymers including PS-*b*-PEO and poly(ethylene-*co*-butylene)-*b*-PEO (KLE) have been often utilized as templates to synthesize mesoporous metal oxides on the basis of the sol–gel reactions^42^,^43^,^44^. In these cases, although organization of micelle hybrids (formed through the interaction between the inorganic precursors and the hydrophilic regions) at mesoscale is demonstrated^42^,^43^,^44^,^45^, the solvent evaporation primarily induces the pre-formed ordered mesophases, where the formation of inorganic frameworks are guided steadily. Our approach is based on ‘micelle assembly’ induced by electromotive force, in which both the metal deposition (that is, the framework construction) and the micelle assembly occur simultaneously, because the electrochemical deposition process of spherical composite micelles is completely proceeded in liquid phases without evaporation of the solvents. Thus, our approach is conceptually different from the previous works on the fabrication of mesoporous films based on the evaporation-induced self-assembly approach. To confirm the role of micelles more clearly, the mesostructure evolution was investigated by scanning electron microscope (SEM) at the initial stages (that is, 10, 30 and 100 s). After 10 s, a large amount of initial Au seeds were formed, as indicated by arrows in Supplementary Fig. 1a. The curved surfaces of the Au branches come from the micelle-directing effect, as indicated by the circle in Supplementary Fig. 1a). When a deposition time was further increased to 30 s, a larger number of mesopores were observed (Supplementary Fig. 1b), though the formation of each mesopore was not perfectly completed. When the deposition proceeded for 100 s, the estimated film thickness reached almost half of the one mesopore size and mesoporous Au framework was well constructed (Supplementary Fig. 1c).

### Characterization of mesoporous Au films

**Figure 2:**
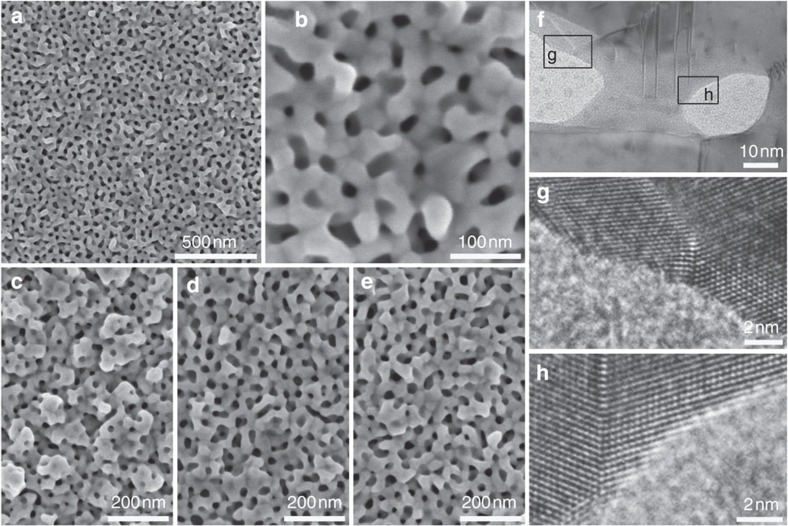
Microscopic characterization of mesoporous Au films. (**a**,**b**) Top-surface SEM images of mesoporous Au film prepared with a typical electrolyte containing PS_18000_-*b*-PEO_7500_ micelles and 3 ml THF as solvent. The deposition time is 1,000 s. (**c**–**e**) Top-surface SEM images of mesoporous Au films prepared with three electrolytes containing PS_18000_-*b*-PEO_7500_ micelles and different THF amounts ((**c**) 1 ml, (**d**) 2 ml and (**e**) 3 ml, respectively). (**f**–**h**) Highly magnified TEM images of mesoporous Au film prepared with a typical electrolyte containing PS_18000_-*b*-PEO_7500_ micelles and 3 ml THF as solvent. The assignment of crystal lattices is shown in Supplementary Fig. 5.

After complete removal of the PS-*b*-PEO micelles, mesoporous Au films were obtained and characterized carefully. Cage-like uniform mesopores were observed in the entire area of the film and connected with one another (Fig. 2a,b). The SEM image of a top surface of the Au film exhibited the presence of uniformly sized mesopores (average 25±5 nm) with wall thickness of 25 nm±5 nm (Fig. 2a,b and Supplementary Fig. 2c). The resultant pore size of the Au film (Fig. 2a,b) was almost the same as the size of spherical micelles observed by TEM (Fig. 1b,c) and SEM (Supplementary Fig. 3), meaning that the spherical micelles serve as the porogens. In addition, pore size in Au film was controllable very easily by changing the amount of THF, herein from 1ml to 2 ml and 3 ml, which is the most important technique related to the tuning of newly proposed optical property arising from concave Au surfaces. Because THF is a good solvent for the PS block, the PS cores in the micelles gradually shrink with the decrease in the amount of THF, thereby reducing pore size of the Au films. Actually, the pore size was controlled down to ~19 nm by decreasing the amount of THF without significant change of the wall thickness (Fig. 2c and Supplementary Fig. 2a). While using the minimum amount of THF (1 ml) for complete dissolution of PS-*b*-PEO, the polymeric micelles, in shrink form, can stably keep the original form and then the resultant mesopores were indeed in spherical form (Fig. 2c). The average pore size and wall thickness are ~19 nm and ~25 nm, respectively. On the other hand, the pore size can be expanded by adding hydrophobic organic compounds. When 1,3,5-triisopropylbenzene (1,3,5-TIPBz) was used as a swelling agent, the effective pore size was increased from 25 nm (without 1,3,5-TIPBz) to 32 nm (1,3,5-TIPBz; 10 μl), 32 nm (20 μl), 40 nm (30 μl) and 40 nm (40 μl; Supplementary Fig. 4). With the increase of the pore size, the pore walls started showing the appearance of protrusions. In addition, the molecular weight of the PS block in PS-*b*-PEO can also be used to control the pore size (Supplementary Fig. 5). As estimated by SEM, the average pore size of a mesoporous Au film prepared using PS_63000_-*b*-PEO_26000_ was around 60 nm.

Unlike sol–gel-based fabrication process of ordered mesoporous metal oxide films, electrochemical deposition provides us the advantage of the facile and precise control of the film growth rate, that is, it enables us to fine tune the film thickness by simply adjusting the deposition time. Film thicknesses of the mesoporous Au films prepared using PS_18000_-*b*-PEO_7500_ for deposition time of 600, 1,000, 1,800, 2,500 and 3,600 s were, respectively, 70, 140, 170, 230 and 440 nm; in this case the average growth rate was calculated to be 0.11 nm s^−1^ (Supplementary Fig. 6). The electrochemically active surface areas (ECSAs) were also calculated by using cyclic voltammetry in an acidic medium (0.5 M H_2_SO_4_) (Supplementary Fig. 7). The peak area indicated by a dotted circle is associated with reduction of Au oxide species (Supplementary Fig. 7a). On the basis of the assumption that the charges associated with the reduction of oxide species is 400 μC cm^−2^ for Au surface 46,47, the ECSAs in the films were theoretically calculated from the observed charges. The total surface area of the mesoporous Au films was increased proportionally (Supplementary Fig. 7b). The volume-normalized ECSAs (per film volume, cm^3^) were around 49.1 m^2^ cm^−3^ (Supplementary Fig. 7c) and almost constant until the film thickness reached 5 μm. Thus, inner parts of the mesoporous Au films can also work as electrochemically active surfaces and it is proved that the mesoporous structures are homogeneously formed inside the films.

To carefully investigate the atomic structure of Au in the pore walls comprehensively, a monolayer of mesoporous Au film was prepared and then its cross-sectional sample was observed by TEM (Fig. 2f–h and Supplementary Fig. 8). The images revealed that spherical mesopores were surrounded by continuous networks of crystalline Au. The lattice fringes associated to Au *fcc* crystal were clearly observed inside the pore walls. High-resolution TEM image taken along <110> directions (Supplementary Fig. 8) showed (111) lattice fringes with stacking faults related to single crystallinity of the mesoporous Au film. Several crystal domains were connected continuously to create concave surface, which exposed high index planes (Fig. 2f–h). There were also several defects such as (i) stacking fault, (ii) dislocation and (iii) kink band, marked by arrows in Supplementary Fig. 8a. These defects could be caused by the localized strains during crystal growth of Au and/or electrochemical deposition of mesostructured films with reduction of soluble Au species. X-ray photoelectronic spectroscopy was used to determine surface composition and oxidation state of the Au film. The high resolution Au 4*f* scan displayed a doublet at 87.7 and 84.0 eV separated by 3.7 eV due to spin–orbit coupling, confirming the presence of Au(0) species in the film (Supplementary Fig. 9).

To realize well-developed mesoporous structures, the crystal growth speed, that is to say, the resultant crystal size, is the most important factor. Wiesner and colleagues have found that diameter of nanoparticles as building blocks should be below a critical limit relative to the sizes of blocks with which they interact^23^. In case of mesoporous metal oxides, mesoporous structure is well preserved only when the wall thickness is larger than the crystal size, which is controlled by the nucleation and grain growth rates^44^. In our electrochemical process, the control of Au crystal sizes is governed by Au growth speed. The bath temperature for the deposition is one of critical factors for the control of Au growth speed. From the amperometric plots for the deposition of mesoporous Au films under different temperatures, it was proved that the reduction currents increased (that is, the deposition rates increased) with the increase of bath temperature (Supplementary Fig. 10). Obviously, the formation of mesoporous structures was confirmed, only when the temperature was <25 °C (Supplementary Fig. 11). In contrast, when a temperature was >40 °C, the Au crystal growth rate was high so that large bulk Au crystals were mainly formed and mesoporous structures were not well developed.

### Optical performance of mesoporous Au films

To investigate the fundamental optical properties of the mesoporous Au films, three typical films with different pore sizes were compared. Here we selected mesoporous Au films prepared using PS_18000_-*b*-PEO_7500_ micelles with different solvent composition (Sample I; 1 ml of THF (average pore size; 19 nm), Sample II; 3 ml of THF (25 nm), Sample III; 3 ml of THF with 40 μl of 1,3,5-TIPBz (40 nm)). The representative scattering spectra were measured by using a custom-made dark-field spectroscopic microscopy (Supplementary Fig. 12), and compared with a spectrum taken from a sputtered Au film without mesopores. As observed in Supplementary Fig. 12b, the scattered light intensity increased systematically with the increase in the pore size, indicating a significant increase of the interaction of the mesopore surfaces with electromagnetic waves in the visible frequency region. The result suggests that the interaction between the Au surface and the light become stronger. This is possibly due to the larger electromagnetic field around the individual mesopores because the charge induced at the surface of the pore becomes larger as the pore size becomes larger as the Au volume exposed to the E-field of the incoming light becomes larger. From another point of view, the reason may be related that expansion of the pores makes the pores respond more efficiently to the light because the pore sizes become closer to the wavelength of the oscillating charge density waves on the Au surface induced by the visible light (Note that the wavelengths of the charge density waves of (localized) surface plasmons are in the sub-100 nm range.). As seen in Supplementary Fig. 12c, each point on the Au surfaces responded in different manner with different scattering intensity as well as different resonance frequency reflecting their various morphologies in nanometer scale. Thus, as shown in Supplementary Fig. 12b, broadband scattering spectra are due to the ensemble effect for the mesopores in the film.

**Figure 3:**
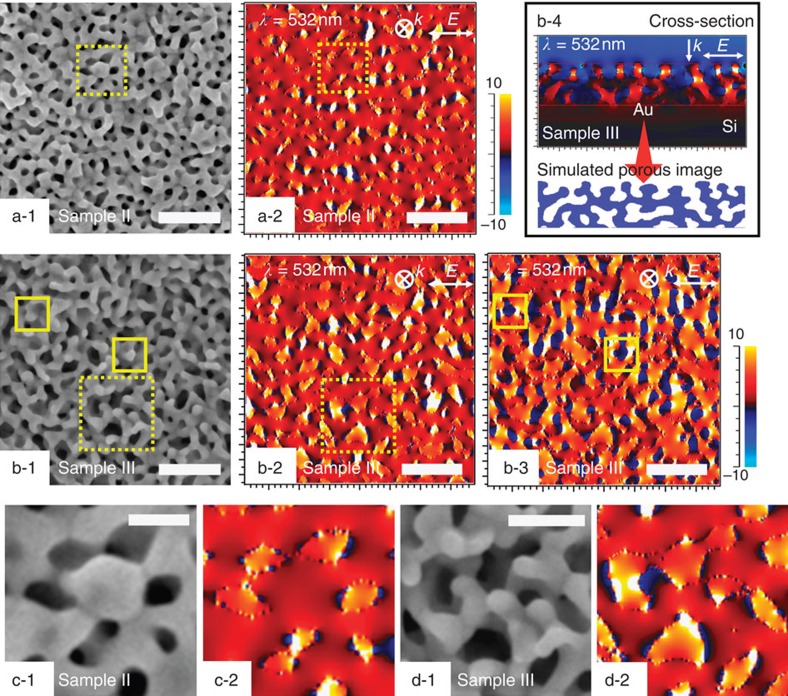
E-field distributions on mesoporous Au films. (**a-1**,**b-1**) SEM images and (**a-2**,**b-2**,**b-3**) the corresponding E-field distributions on mesoporous Au films prepared with two electrolytes containing PS_18000_-*b*-PEO_7500_ micelles with different solvent compositions ((Sample II) 3 ml THF, and (Sample III) 3 ml THF+40 μl 1,3,5-TIPBz) under 532 nm excitation. The E-field distribution in **a-2** and **b-2** is taken from 10 nm in depth in the films, in which moderate E-field amplitude is clearly observed inside or at the perimeter of the mesopores, as shown in dotted-line squares. The E-field distribution in **b-3** is taken from the film surface, showing strong E-field points (hot spots) at the perimeter of the protruded objects (as marked by solid-line squares). (**b-4**) Cross-sectional E-field distributions on mesoporous Au film (Sample III). (**c,d**) Enlarged SEM images and the corresponding E-field distributions of the areas indicated by dotted-line squares of **a** and **b**. Scale bars, 200 nm (**a**,**b**), 50 nm (**c**) and 100 nm (**d**), respectively.

**Figure 4:**
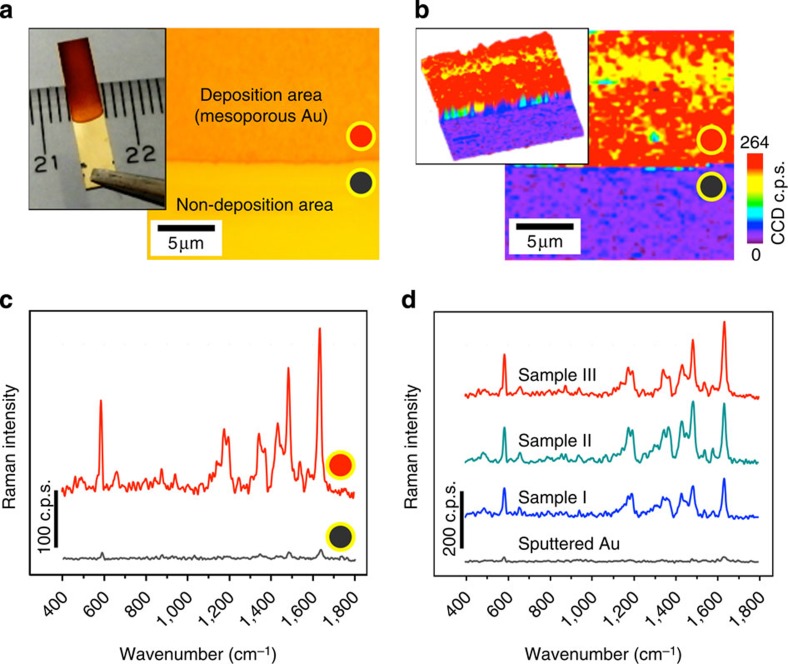
SERS study of Nile blue-molecules coated on mesoporous Au films. (**a**) Photograph and optical image of mesoporous Au film (Sample III) prepared with an electrolyte containing PS_18000_-*b*-PEO_7500_ micelles and 3 ml THF+40 μl 1,3,5-TIPBz as solvent. (**b**) Corresponding SERS spectral mapping with vibrational intensity. (**c**) Representative SERS spectra on mesoporous Au film (Sample III). Mesoporous Au region (that is, deposition area) and non-porous region (that is, non-deposition area) are measured, respectively. (**d**) Representative SERS spectra on mesoporous Au films prepared with three electrolytes containing PS_18000_-*b*-PEO_7500_ micelles with different solvent compositions ((Sample I) 1 ml THF, (Sample II) 3 ml THF and (Sample III) 3 ml THF+40 μl 1,3,5-TIPBz). A sputtered Au film without mesopores is also compared. The variability of the SERS spectra is ±5% for each, which is due to the variability of the surface roughness and the uniformity of the adsorbed molecular layers.

To gain deeper insight into the observed spectra, E-field distribution was assessed on the basis of simulations using finite-difference time domain (FDTD) algorithm. As clearly seen, experimentally observed tendency of the scattering intensity of the three films (Supplementary Fig. 12) had clear correspondence to the strength of the E-field in Fig. 3 and Supplementary Fig. 13. The E-field amplitude increased as the pore size increased (Supplementary Fig. 13). Interestingly, moderate E-field amplitude was clearly seen inside or at the perimeter of the mesopores (Fig. 3c,d). For larger pores on the contrary, there was a clear tendency that strong E-field points (hot spots) emerge at the perimeter of the protruded objects, as clearly seen in Fig. 3b-3. The cross-sectional image also confirmed the above characteristic, which showed the outbreak of the strong E-field inside the mesopores (Fig. 3b-4). Especially for the Sample III, the pores become agglomerated with connected meandering pore walls with prominent nanosized protrusions and the E-field has a tendency to be converged there giving high amplitude (Fig. 3b-3). Such nanoprotrusions on elongated walls show plasmon resonance frequency in the longer wavelength (near infrared) region. In contrast, the other two films (Samples I and II with smaller pores) have less corrugated surface and less protrusions, resulting in slightly higher resonance frequency (that is, shorter wavelength) and smaller E-field as well as scattering.

Stability of the mesoporous Au film in water and strong affinity of the Au surfaces to proteins and amino acids are helpful for utilizing the films as the perfect substrate for biosensing. Most of the state-of-the-art apta-sensing techniques are often based on thiolated aptamers with rationally designed linker molecules for the specific target molecules. Then, it is considered that the present mesoporous Au films are the best substrate suitable for SERS for apta-sensing. Here we examined the SERS performance for the above three typical films (Samples I, II and III) with different pore size (Fig. 4). A droplet (5 μl) of Nile blue solution (10^−6^ M in ethanol) was spread uniformly on the mesoporous Au films (0.3 cm × 1.5 cm) and the substrates were dried in air at room temperature under a stream of nitrogen gas. The SERS spectra, taken from Nile blue molecule coated on mesoporous Au films, with laser excitation wavelength of 532 nm were recorded. Optical microscope image and corresponding SERS spectral mapping images for Sample III are shown in Fig. 4a–b. It was clearly visualized that the SERS signal was drastically enhanced on mesoporous Au region. The enhancement mechanism of SERS effect is mainly due to the field enhancement effect arising from the mesoporous Au. The sputtered Au surface without mesopores shows far less SERS signal intensity. The most dramatic effect that arises from the nanomorphology is indeed a field enhancement induced by the strong-light Au surface interaction, as can be seen from the enhanced light scattering (Supplementary Fig. 12b) and thus we can assign this SERS effect as electromagnetic origin (Fig. 3 and Supplementary Fig. 13). In addition, the chemical SERS effect induced by the molecule adsorption should be minor, because its contribution to the SERS intensity is in the order of the black spectrum in Fig. 4c where the electromagnetic field amplitude is substantially reduced in comparison with mesoporous Au films. Furthermore, the SERS chemical effect is a local chemical bond effect, which is determined by the local bond nature between the Nile blue molecules and the Au surface. Therefore, we can safely conclude that the difference in SERS intensity is attributed to the surface morphology effect, as the adsorbed Nile blue should be multilayered.

Furthermore, the SERS signal intensity from the molecule was increased with the increase in the mesopore size (Fig. 4d). This result exhibits the same tendency as the scattering intensity shown in Supplementary Fig. 12b, reflecting the difference in the pore architecture of the films. This result is understandable because the stronger light scattering points to the generation of enhanced E-field at the pore surfaces caused by the excitation of localized surface plasmon. Then, this enhanced E-field can subsequently lead to a stronger SERS signal from the adsorbed molecules, as observed in our measurement (Note that a stronger SERS intensity is expected for the laser excitation wavelength around 670 nm close to that of the peak position in the scattering spectra. However, our experiment using a 532 nm laser has already showed enough SERS signal which evidences the high performance of this mesoporous Au substrate.). To estimate the SERS enhancement factor, Au sputtered film (that is, non-SERS substrate) with the same area was coated with a droplet (5 μl) of Nile blue solution with a high concentration of 10^−3^ M in ethanol (to visualize the non-SERS weak signals, Fig. 4c). Using the same measurement conditions, the SESR enhancement factor is simply calculated by the following formula^48^.


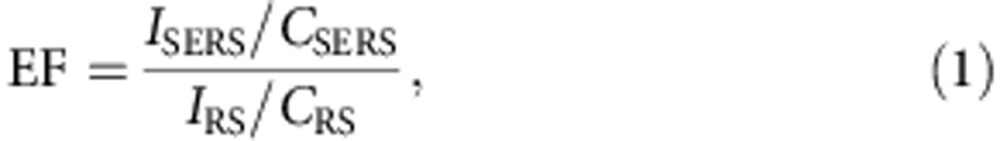


where *I*_SERS_ and *I*_RS_ are Raman intensities of the SERS and non-SERS signal, whereas *C*_SERS_ and *C*_RS_ are concentrations of the dropped molecule SERS and on the SERS and non-SERS substrate, respectively. The intensity of the strongest peak at 1,642 cm^−1^ was used for the calculation, leading to the calculated enhancement factor of 1.2 × 10^5^. The signal enhancement effect of our materials is in the similar order as other nanostructured samples previously reported^49^,^50^,^51^, but the use of mesoporous Au substrates successfully guides to the systematic understanding for correlating the local optical properties and the fine-tuned morphologies by using spectral mapping with high spatial resolution. Such information is definitely useful for elucidating the origin and mechanism of electromagnetic field enhancement.

To further demonstrate the large-sized mesopores, we show selective detection of large biomolecules (protein molecules) on our mesoporous Au film (Supplementary Fig. 14). SEIRA was carried out using Fourier transform infrared (FTIR) configuration in attenuated total reflection geometry. For this measurement, a self-assembled monolayer of bovine serum albumin (BSA, the average diameter of 8.6 nm) adsorbed on the cleaned mesoporous substrate was used as a target protein. Mesoporous Au film (Sample II, 0.3 cm × 1.5 cm) was soaked into BSA solution (10^−3^ M in water, pH=7.1) for 24 h, then thoroughly rinsed in distilled water to leave a monolayer of BSA on the pore surface, and finally dried under a stream of nitrogen gas. As shown in Supplementary Fig. 14, the protein bands (Amide-I and -II) are clearly observed evidencing the adsorption of monolayer of BSA. Very high signal intensity of self-assembled monolayer-BSA protein is observed on our mesoporous Au film. Note that the enhancement factor scales as the (*E*/*E*_i_)^2^ for SEIRA, unlike the (*E*/*E*_i_)^4^ for the SERS, in the visible frequency region (Here *E* means local electrical field on the mesoporous Au substrate and *E*_i_ means electrical field of the incident light.). Considering the above difference and also the smaller electromagnetic field enhancement in the infrared compared with the visible region of this sample, the signal enhancement of the SEIRA shown here is high enough and satisfactory.

## Discussion

We have developed an efficient electrochemical deposition approach to fabricate mesoporous Au films from a diluted electrolyte containing micelles. The electrolyte solutions were studied by ultraviolet–visible absorption and Raman spectroscopy (Supplementary Fig. 15). In the ultraviolet spectra, the peaks at around 324 nm correspond to a typical *d*-*d* transition of AuCl_4_^−^ species. Another band at around 278 nm appears by the presence of benzene rings in the PS block. The Raman spectra display several peaks at 347, 324 and 171 cm^−1^, due to symmetric (A_1g_), antisymmetric (B_2g_) and bending (B_1g_) modes of AuCl_4_^−^ species in the solution, respectively. All other peaks were related to the solvents and the PS-*b*-PEO diblock copolymer. Both the ultraviolet–visible absorption and the Raman spectra show that the Au species are considered as Au(III) with Cl^−^ ligand. Accordingly, in this study, our approach to obtain mesoporous Au films was succeeded through effective interaction between PS-*b*-PEO micelles and soluble Au species; HAuCl_4_ are dissolved into H_3_O^+^ and AuCl_4_^−^ and then interacted with the EO shell domains of the PS-*b*-PEO micelles by hydrogen bonding (Fig. 1a). Moreover, we consider that AuCl_4_^−^ is free ion in the solution as well as in the hydrophilic domains of the PS-*b*-PEO micelles. The micelles with AuCl_4_^−^ can be neutral, negatively and positively charged, depending on the H_3_O^+^/AuCl_4_^−^ ratio in the part of the EO shells. In our experimental conditions, the micelle solution after the addition of aqueous solution of HAuCl_4_ was slightly positive in zeta-potential. Thus, the H_3_O^+^ rich micelles were positively charged and then moved to the working (negative) electrode surface. The AuCl_4_^−^ (redox potential of AuCl_4_^−^/Au^0^ is 0.93 V versus standard hydrogen electrode) species were reduced to metallic Au from the part of near the electrode surface consecutively under a constant potential of −0.5 V. Our approach shows a fairly high reproducibility (100%) (Supplementary Fig. 16). The electrodeposited mesoporous Au films were well adhered on the substrates (that is, working electrodes) (Supplementary Fig. 17).

As demonstrated above, the present electrochemically deposited mesoporous Au films with tunable pores can provide fascinating optical properties tailored by engineering the soft-templated uniform mesospaces. Although several efforts have been made for preparing mesoporous Au films, continuous Au films with precisely designed mesopores have not been realized yet because of the difficulty in controlling the crystal growth of Au. To overcome this issue, electrochemical synthesis utilizing stable micelles of PS-*b*-PEO diblock copolymers as pore-directing agents is quite effective. In the resultant films, uniformly sized mesopores are distributed in the entire films and controlled in a wide range from 20 to 60 nm by changing the molecular weight of PS-*b*-PEO and the electrolyte composition. Our approach not only demonstrates novel optical applications of mesoporous Au films but also sheds new light on the probability of obtaining mesoporous metals through the soft-templating pathway. Such 3D extended metallic frameworks can steadily provide abundant adsorption and reaction sites of target molecules, which are critical to realize emergent functions in the future. Our electrochemical approach is widely applicable to embed uniform mesopores in other metal and alloy systems, which are generally difficult to be synthesized.

## Methods

### Preparation of mesoporous Au films

Our approach is based on an electrochemical method, which has been practically used for the general preparation of continuous metallic films. In a typical synthesis, 10 mg of polystyrene-*b*-poly(oxyethylene) (PS-*b*-PEO) was dissolved in 3 ml of THF completely at 40 °C and then 1.5 ml of ethanol was added to the solution. An aqueous solution of HAuCl_4_ (the final concentration was 5 mM in 8 ml of electrolyte) was added slowly to the clear PS-*b*-PEO solution and spherical micelles were formed by the presence of water. Gentle stirring for 30 min at room temperature is used to make sure that the dissolved Au species were well incorporated into the exterior PEO region of the micelles. Finally, a transparent bright-yellow coloured electrolyte (pH=2.5) was obtained and directly used for electrodeposition. Electrochemical deposition from the precursor solutions was carried out by using an electrochemical machine (CHI 842B electrochemical analyzer, CH Instrument, USA) with a conventional three-electrode system, including a platinum wire as a counter electrode, an Ag/AgCl as a reference electrode and a conducting substrate as a working electrode. The schematic illustration of the typical electrodeposition cell is shown in Supplementary Fig. 18. In this work, the typical conducting substrate used is Au–Si wafer with a representative size of 0.45 cm^2^ (0.3 cm × 1.5 cm), which was fabricated by a dicing cutter. The optimal electrodeposition of Au was carried out at a constant potential of −0.5 V (versus Ag/AgCl) for 1,000 s without stirring at room temperature. During the electrodeposition, a stable current was detected for the Au reduction, as displayed in Supplementary Fig. 10. After the Au deposition, the micelles (used as soft-templates) were thoroughly removed by ultraviolet–ozone cleaner or low-powered O_2_ plasma treatment, as confirmed by infrared analysis. Calcination of the films in air, which has been commonly used for complete removal of organic templates in mesoporous metal oxide films, led to removal of the templates from the films, but the mesoporous structures collapsed through the grain growth of Au.

### Characterization

SEM images were obtained using a Hitachi HR-SEM SU8000 microscope at the accelerating voltage of 5 kV. Transmission electron microscope (TEM) images were taken by using a JEOL JEM-2100F microscope at the accelerating voltage of 200 kV. X-ray photoelectronic spectroscopic measurements were conducted by using a JPS-9010TR (JEOL) instrument with an Mg Kα X-ray source. The precursor solutions were studied by a JASCO V-570 ultraviolet–visible–near infrared spectrometer. The Raman spectra were recorded by a Horiba-Jobin-Yvon T64000 Raman spectroscopy system with the laser wavelength of 514.5 nm. The electrodeposition of mesoporous Au films and cyclic voltammetry measurement were performed by using a CHI 842B electrochemical analyzer (CH Instruments). A conventional three-electrode cell was used, including an Ag/AgCl electrode as reference electrode, a platinum wire as counter electrode and a working electrode. The scattering spectra and SERS properties were carried out using a custom-made confocal Raman microscope (WITec Alpha 300S) combined with a monochromator (Action SP2300—Princeton Instruments) and a CCD camera (Andor iDus DU-401A BR-DD-352). Both spatial resolution of dark-field and SERS scanning were estimated to be ~300 nm (64 × 64 pixels on a 20 × 20 μm scanning area) with the integration time of 0.2 s per pixel. An ultraviolet–near infrared light source (halogen lamp HAL 100-Zeiss) and a dark-field lens ( × 50/0.55 NA-Zeiss) were used to study the dark-field scattering properties. For the characterization of SERS properties, a second harmonic diode pumped Nd:VO_4_ laser (Witec) at 532 nm (0.5 mW) was focused on the scanning area of the Nile blue-treated mesoporous Au films by using a × 100 objective (0.9 NA-Olympus). Before each measurement, the intensity calibration was done carefully by measuring the Si Raman peak intensity of the Si wafer. SEIRA was carried out using FTIR configuration in the attenuated total reflection geometry (Nicolet IS50R-FTIR). The electric field distributions of the Au mesoporous structures were calculated using 3D finite-difference time-domain method (Fullwave, Rsoft). The 3D model was performed on the basis of geometries of mesoporous Au films. The excited electric field propagating along the *z* axis was injected from the top of the Au mesoporous surface with their electric vectors oscillating along the *xz* plane.

## Author contributions

C.L. synthesized and characterized mesoporous Au films, Ö.D. analysed ultraviolet–visible and Raman data, T.D.D. and T.N. studied mesospace-stimulated optical properties, Y.S. and O.T. analysed TEM data, Ö.D. and T.K. contributed to discussion on formation mechanism and Y.Y. designed this work. All the authors discussed the results and participated in writing the manuscript.

## Additional information

**How to cite this article:** Li, C. *et al*. Electrochemical synthesis of mesoporous Au films toward mesospace-stimulated optical properties. *Nat. Commun.* 6:6608 doi: 10.1038/ncomms7608 (2015).

## Supplementary Material

Supplementary Information Supplementary Figures 1-18 and Supplementary Notes 1-5
