# Supplementary material for: Electrochemical synthesis of mesoporous gold films toward mesospace-stimulated optical properties
Source: Nat Commun. 2015 Mar 23;6:6608. doi: 10.1038/ncomms7608 (PMC4382992; doi:10.1038/ncomms7608)
Supplement: Supplementary Information — Supplementary Figures 1-18 and Supplementary Notes 1-5 [file ncomms7608-s1.pdf]

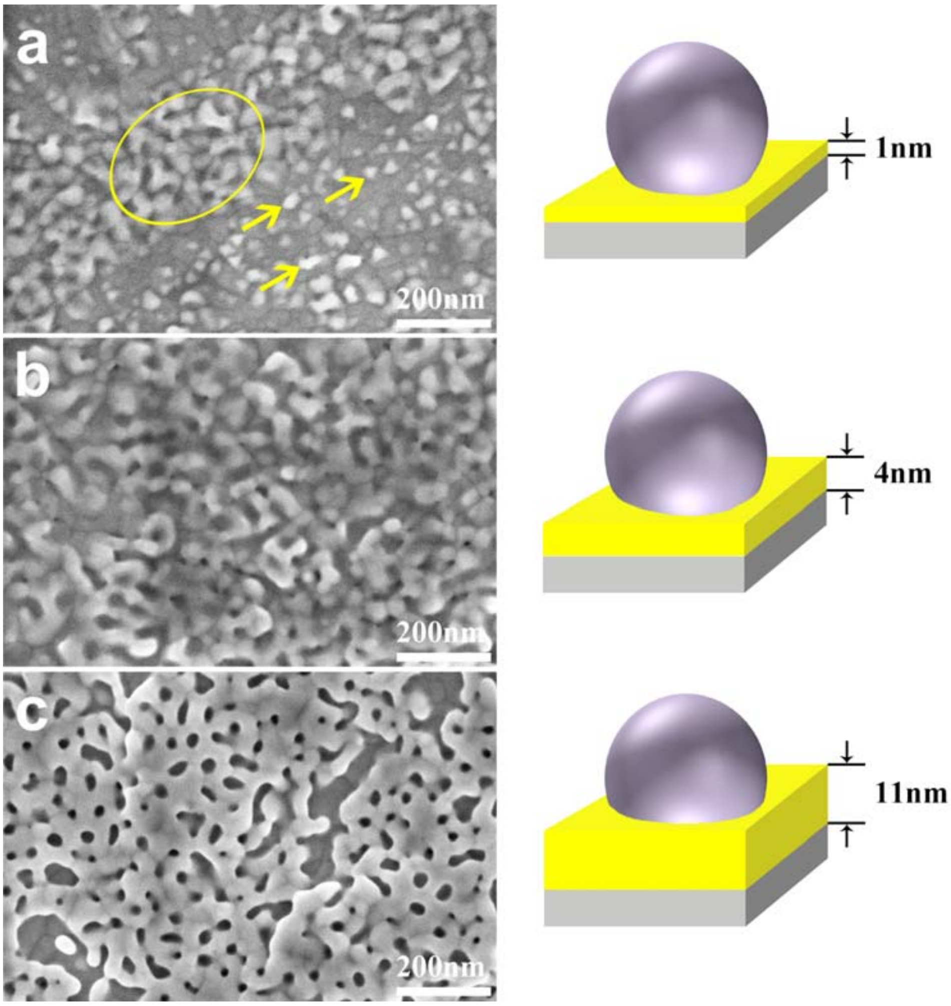

Supplementary Figure 1 | Top-surface SEM images of mesoporous Au films prepared for very short deposition times ((a) 10 s, (b) 30 s, and (c) 100 s). All the films are prepared with a typical electrolyte containing PS_{18000}-*b*-PEO_{7500} micelles and 3 mL THF as solvent.

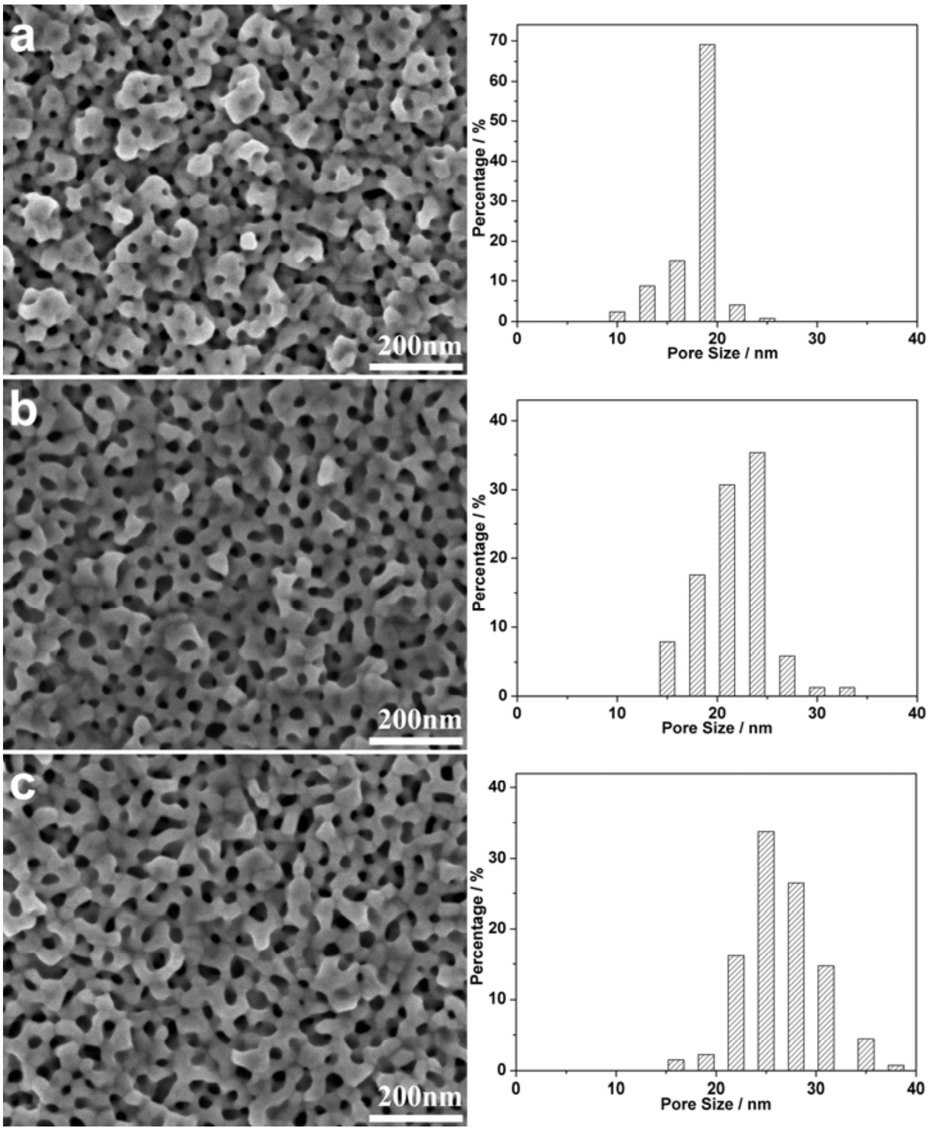

Supplementary Figure 2 | Top-surface SEM images and the corresponding pore size distribution histograms of mesoporous Au films prepared with three electrolytes containing PS_{18000}-*b*-PEO_{7500} micelles and different THF amounts ((a) 1 mL, (b) 2 mL, and (c) 3 mL, respectively). The average mesopore sizes are (a) 19 nm, (b) 24 nm, and (c) 25 nm, respectively.

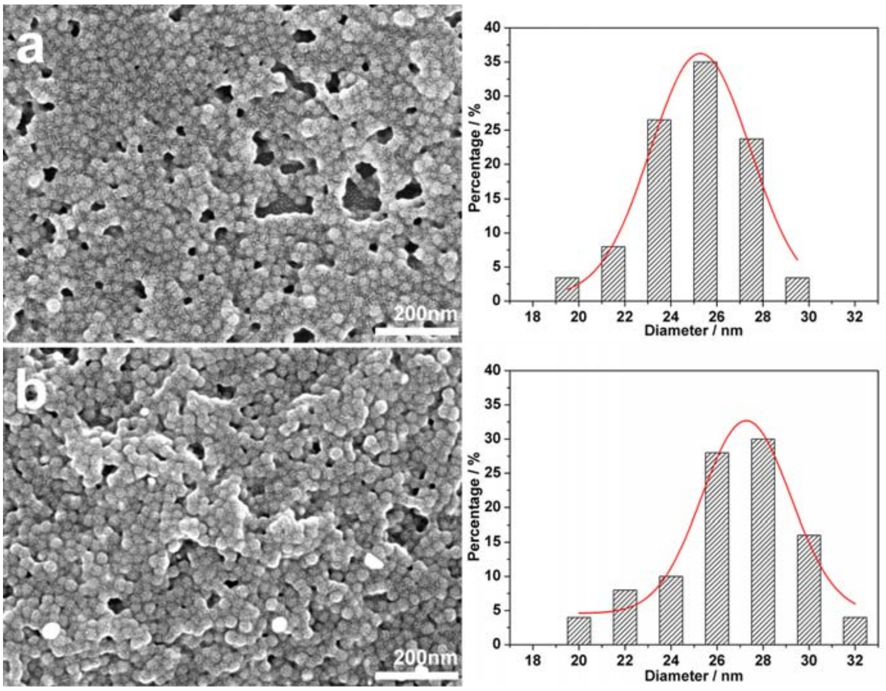

Supplementary Figure 3 | SEM images of PS_{18000}-*b*-PEO_{7500} micelles formed in aqueous solution (with 3 mL THF) (a) without and (b) with $HAuCl_4$ source, and their corresponding micelle size distribution histograms.

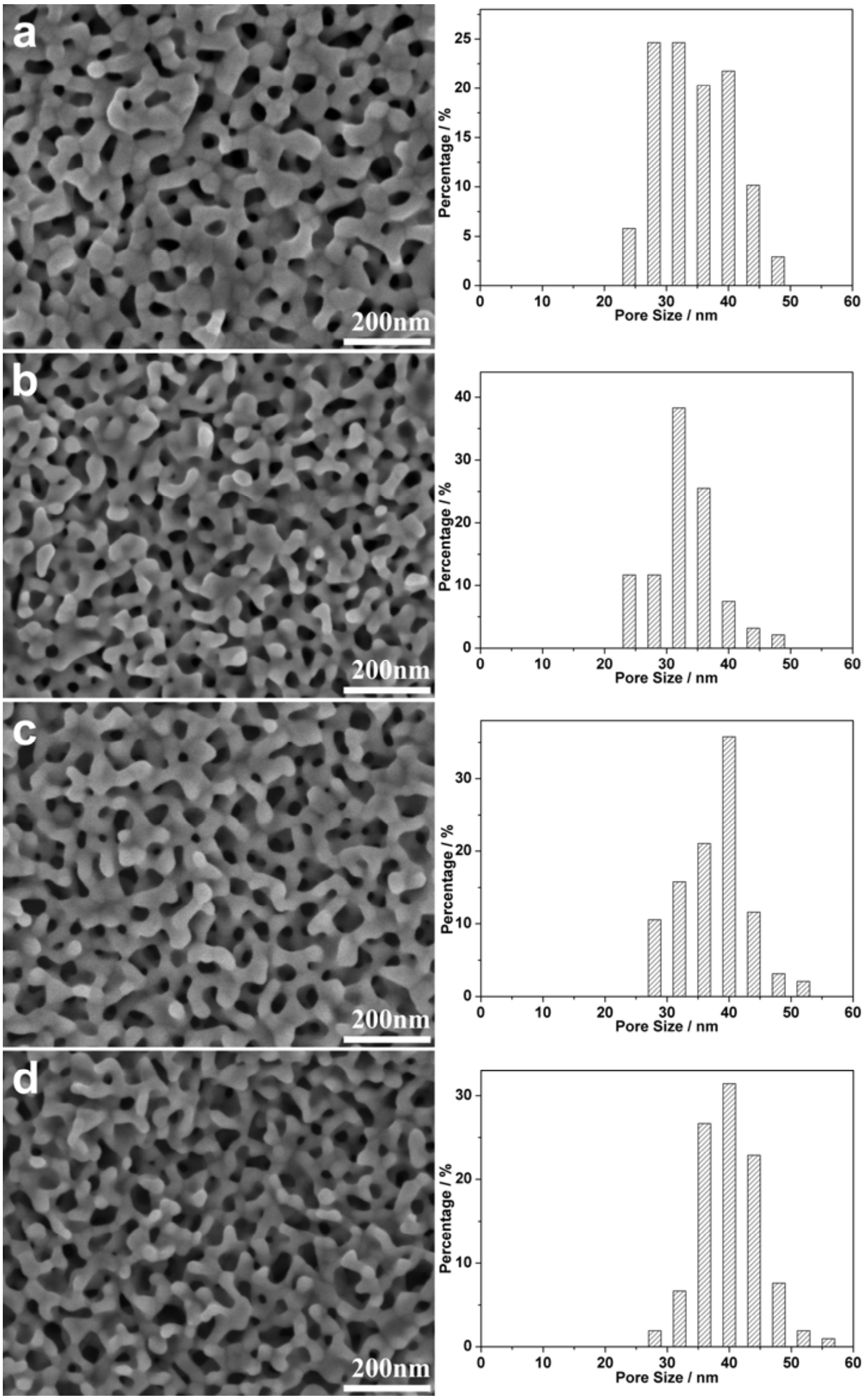

Supplementary Figure 4 | Top-surface SEM images and the corresponding pore size distribution histograms of mesoporous Au films prepared with four electrolytes containing PS_{18000}-*b*-PEO_{7500} micelles and different 1,3,5-TIPBz amounts ((a) 10 μL, (b) 20 μL, (c) 30 μL, and (d) 40 μL). THF amount is fixed to be 3 mL. The average mesopore sizes are (a) 32 nm, (b) 32 nm, (c) 40 nm, and (d) 40 nm, respectively.

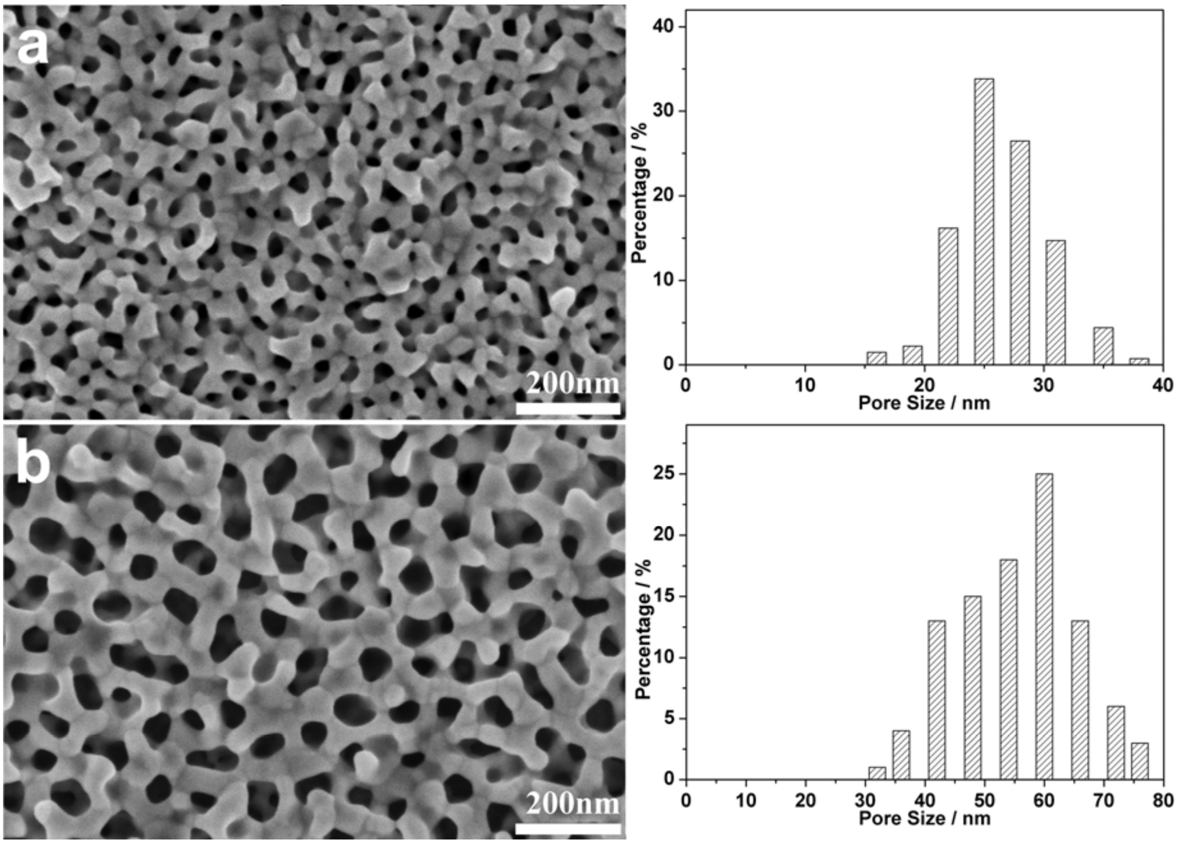

Supplementary Figure 5 | Top-surface SEM images and the corresponding pore size distribution histograms of mesoporous Au films prepared with two different electrolytes containing (a) PS_{18000}-*b*-PEO_{7500} micelles and (b) PS_{63000}-*b*-PEO_{26000} micelles, respectively. THF amount is fixed to be 3 mL. The average mesopore sizes are (a) 25 nm and (b) 60 nm, respectively.

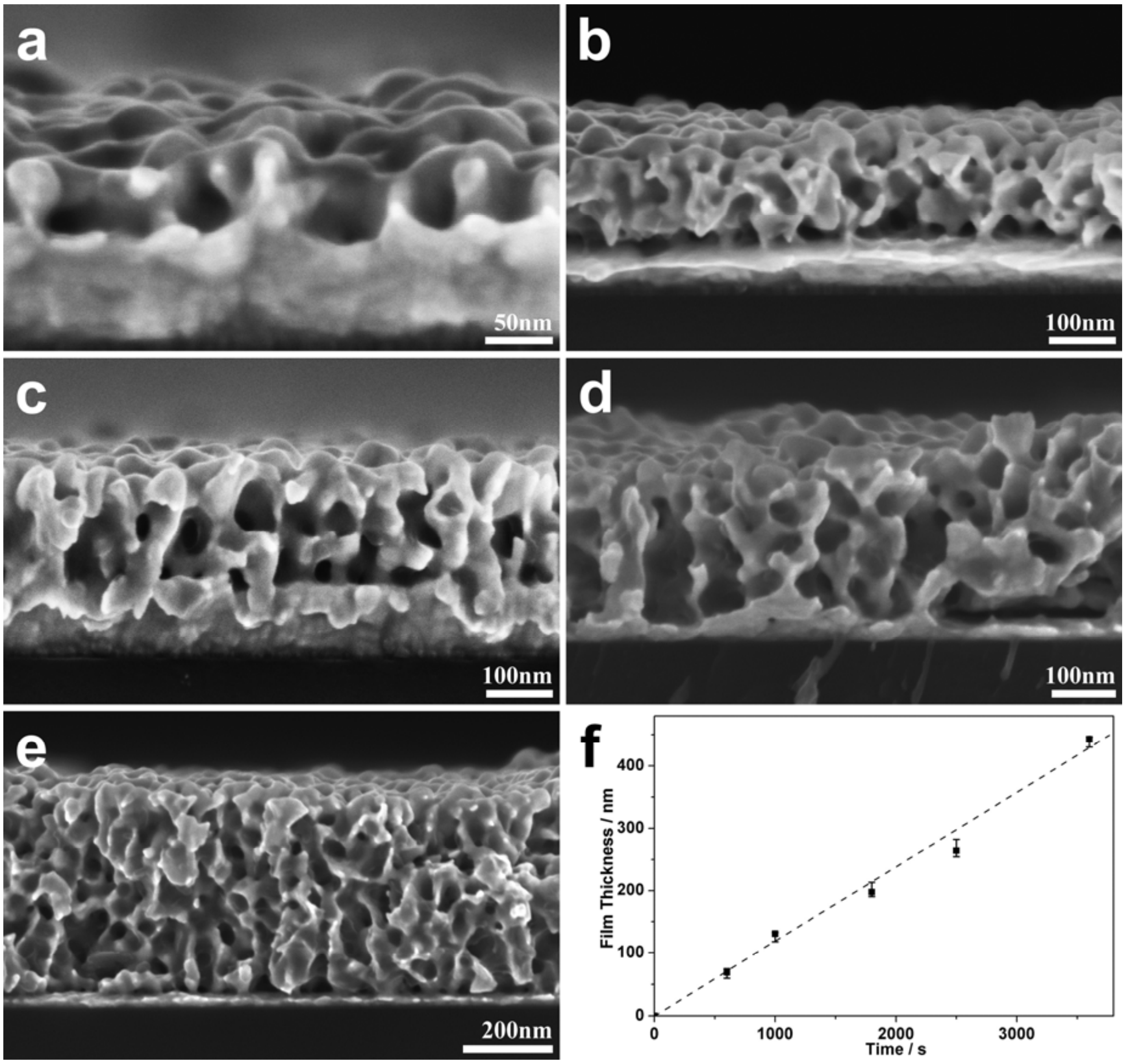

Supplementary Figure 6 | Relationship between the deposition times and the film thicknesses. (a-e) Cross-sectional SEM images of mesoporous Au films prepared with a typical electrolyte containing PS_{18000}-*b*-PEO_{7500} micelles and 3 mL THF as solvent. The deposition times are (a) 600 s, (b) 1000 s, (c) 1800 s, (d) 2500 s, and (e) 3600 s, respectively. (f) Growth rate of mesoporous Au films. The pore shapes are slightly distorted in the cross sections, due to Au characteristic ductility and malleability.

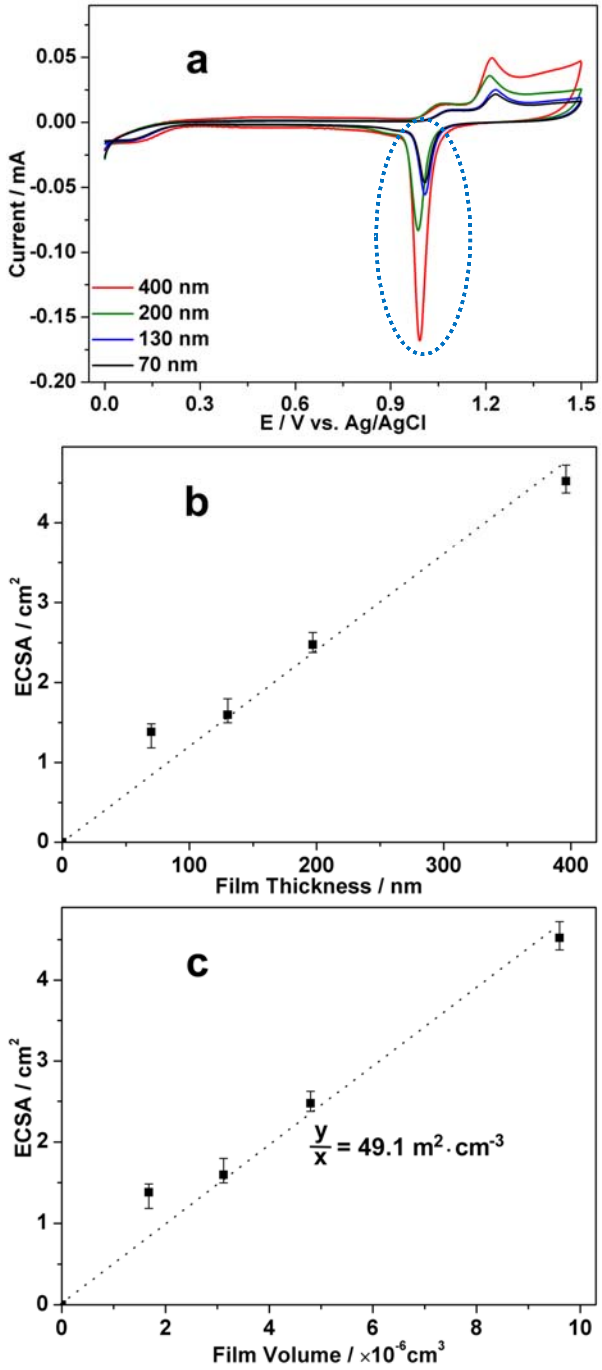

Supplementary Figure 7 | Calculation of ECSAs for mesoporous Au films prepared with a typical electrolyte containing PS_{18000}-*b*-PEO_{7500} micelles and 3 mL THF as solvent. (a) Cyclic voltammograms (CVs) for mesoporous Au films with different film thicknesses in an acidic medium (0.5 M H_2SO_4). (b) Relation between the total ECSAs and the film thicknesses. (c) Relation between the total ECSAs and the film volumes.

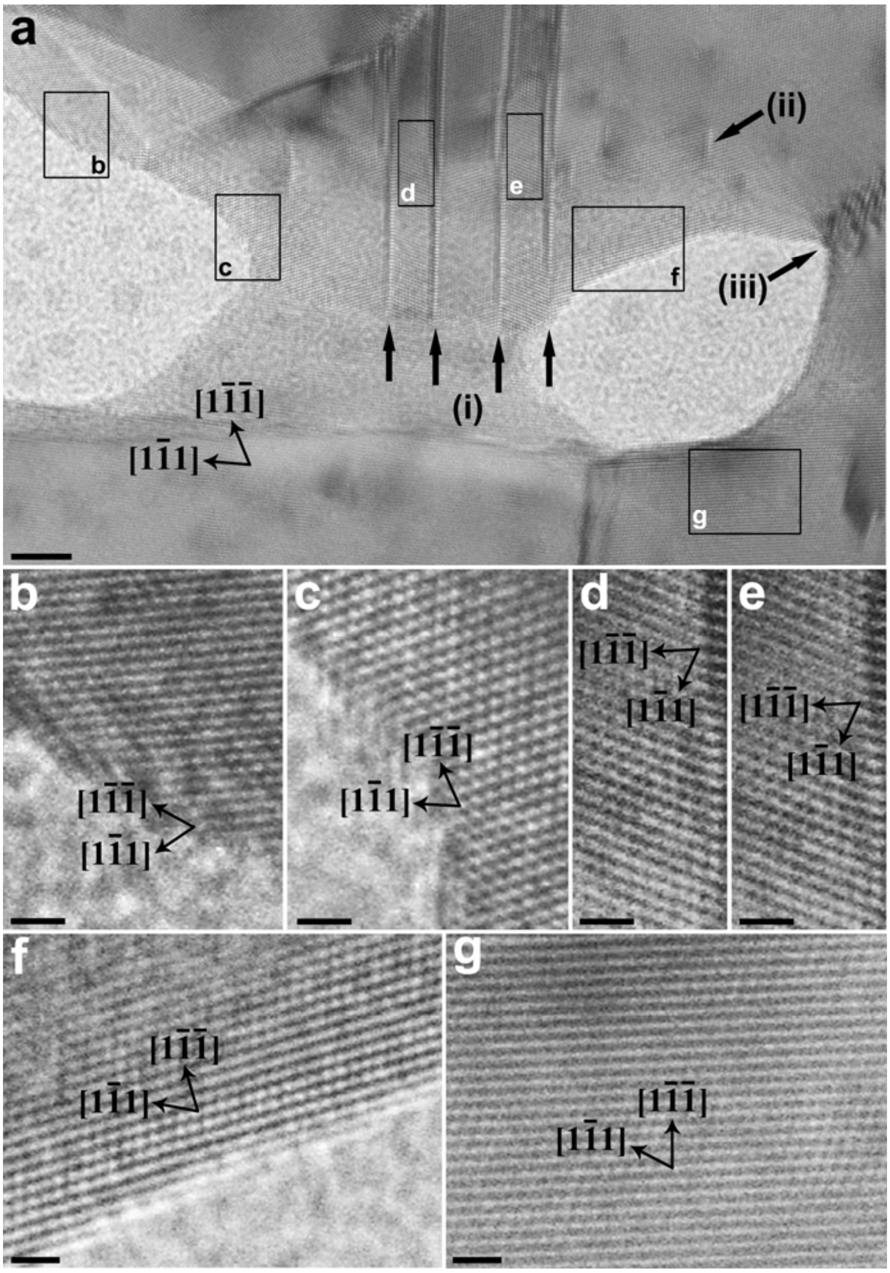

Supplementary Figure 8 | Highly magnified TEM images of mesoporous Au film prepared with a typical electrolyte containing PS_{18000}-*b*-PEO_{7500} micelles and 3 mL THF as solvent. Several defects such as (i) stacking fault, (ii) dislocation, and (iii) kink band, are marked by arrows, respectively. The scale bars are (a) 5 nm and (b-g) 1 nm, respectively. The zone axis is <110> direction.

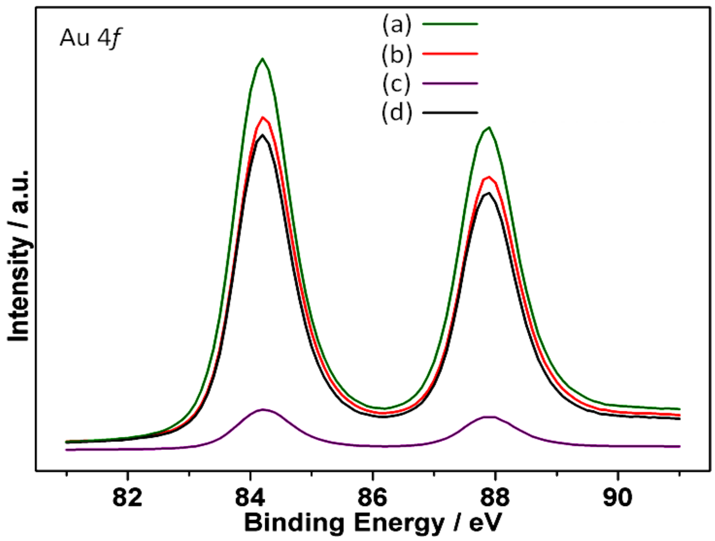

Supplementary Figure 9 | XPS spectra of (a-c) mesoporous Au film prepared with a typical electrolyte containing PS_{18000}-*b*-PEO_{7500} micelles and 3 mL THF as solvent ((a) mesoporous Au film after removal of polymer micelles, (b) mesoporous Au film after an etching treatment (5 nm of thickness is removed from the top surface.), and (c) mesostructured Au film before removal of polymer micelles, respectively.) and (d) nonporous Au film prepared by a sputtering method.

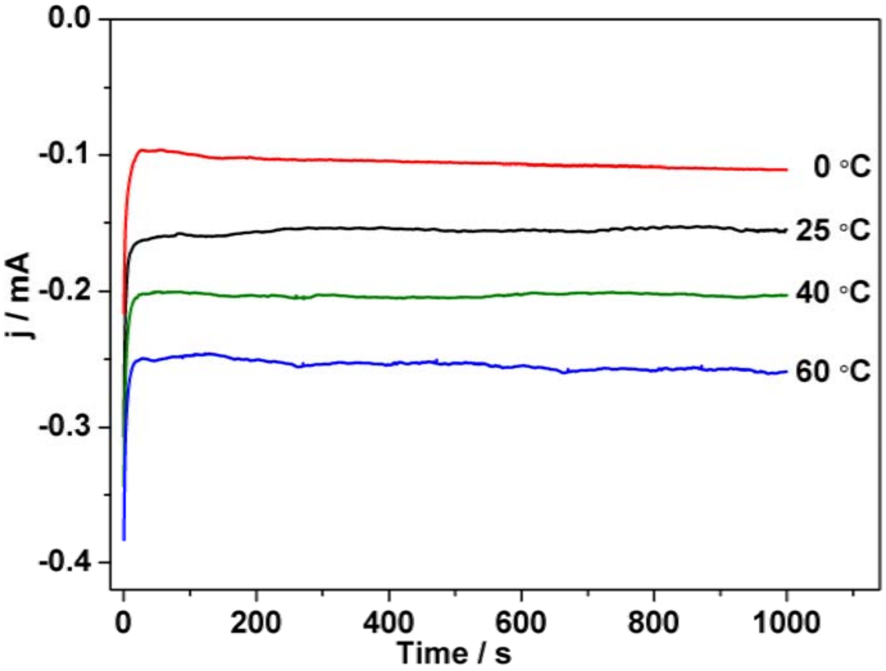

Supplementary Figure 10 | Amperometric plots for deposition of mesoporous Au films under different temperatures. All the deposition processes are carried out under a typical electrolyte containing PS_{18000}-*b*-PEO_{7500} micelles and 3 mL THF as solvent.

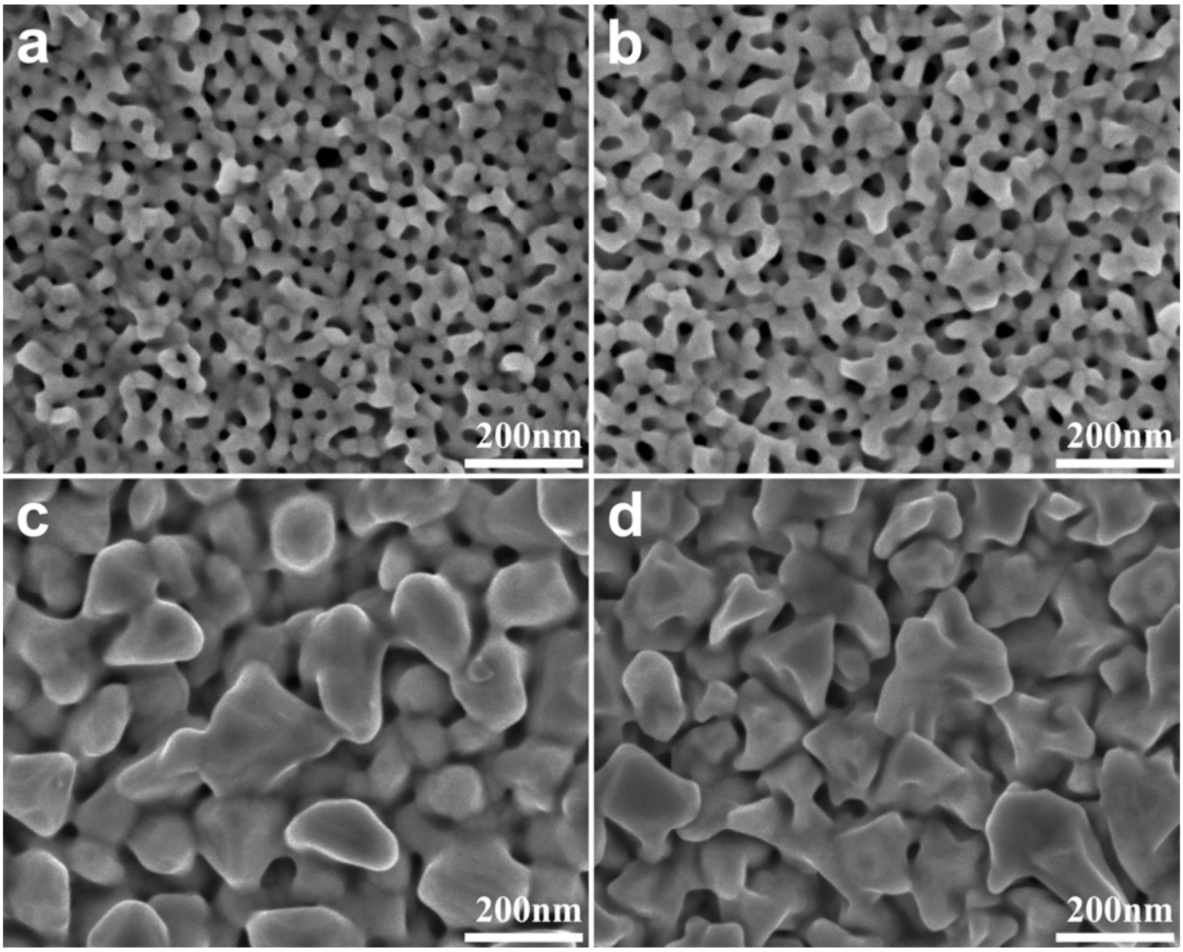

Supplementary Figure 11 | SEM images of Au films prepared at different temperatures ((a) 0 °C, (b) 25 °C, (c) 40 °C, and (d) 60 °C.). All the films are prepared with a typical electrolyte containing PS_{18000}-*b*-PEO_{7500} micelles and 3 mL THF as solvent.

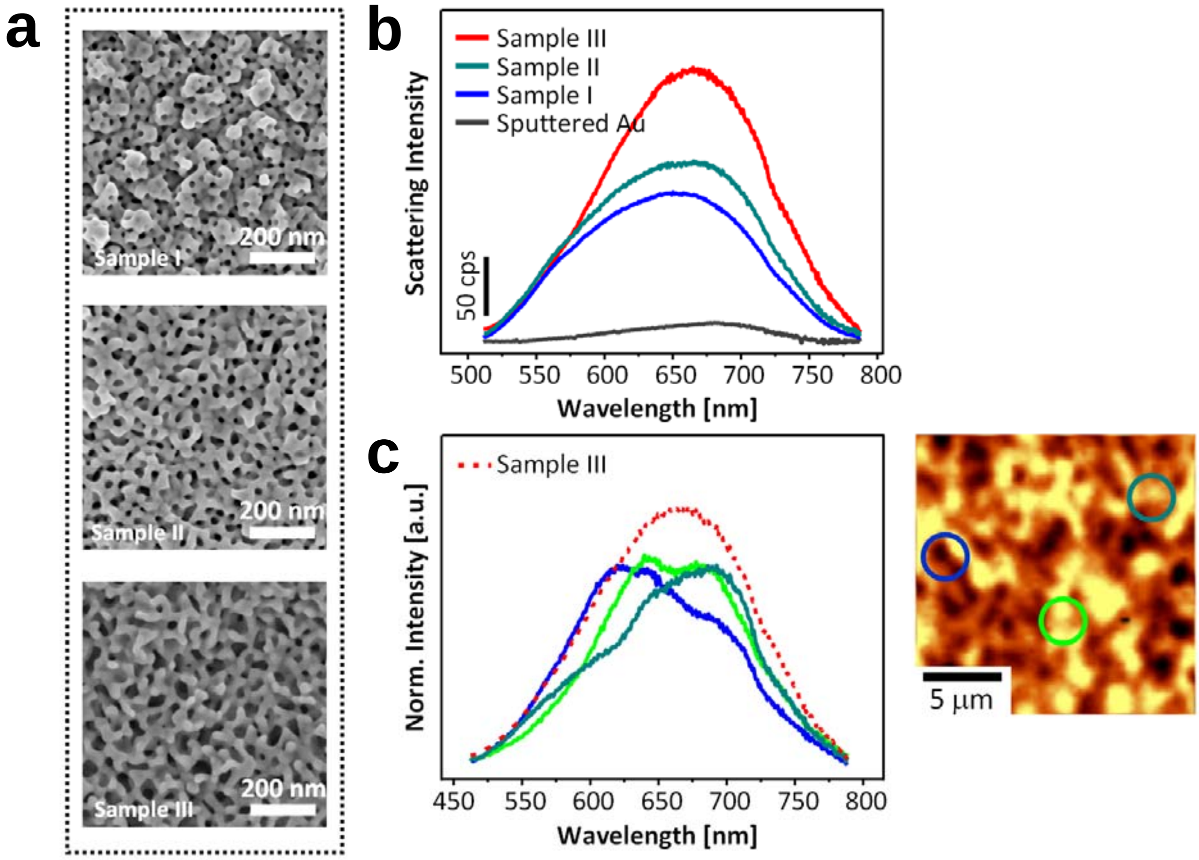

Supplementary Figure 12 | (a) SEM images of mesoporous Au films prepared with three electrolytes containing PS_{18000}-*b*-PEO_{7500} micelles with different solvent compositions ((Sample I) 1 mL THF, (Sample II) 3 mL THF, and (Sample III) 3 mL THF + 40 μL 1,3,5-TIPBz). (b) Scattering spectra of mesoporous Au films (Sample I, Sample II, and Sample III). A sputtered Au film without mesopores is also compared. (c) Position dependence of the scattering spectra of mesoporous Au film (Sample III). Broadband spectrum observed in panel (b) occurs via ensemble effect of the optical response from various different types of mesopores and nanoprotrusions in the film.

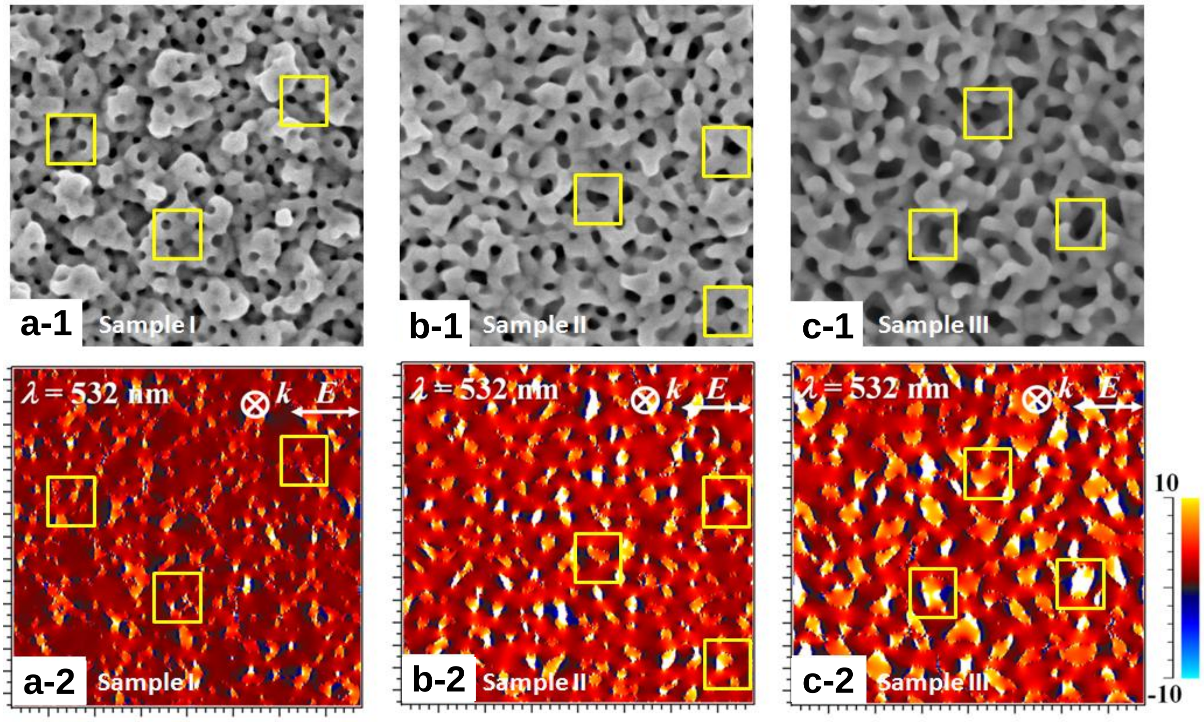

Supplementary Figure 13 | (a-1, b-1, c-1) SEM images and (a-2, b-2, c-2) the corresponding E-field distributions on mesoporous Au films prepared with three electrolytes containing PS_{18000}-*b*-PEO_{7500} micelles with different solvent compositions ((Sample I) 1 mL THF, (Sample II) 3 mL THF, and (Sample III) 3 mL THF + 40 μL 1,3,5-TIPBz) under 532 nm excitation. The E-field enhancement on the mesoporous Au structure increases as the pore size increases.

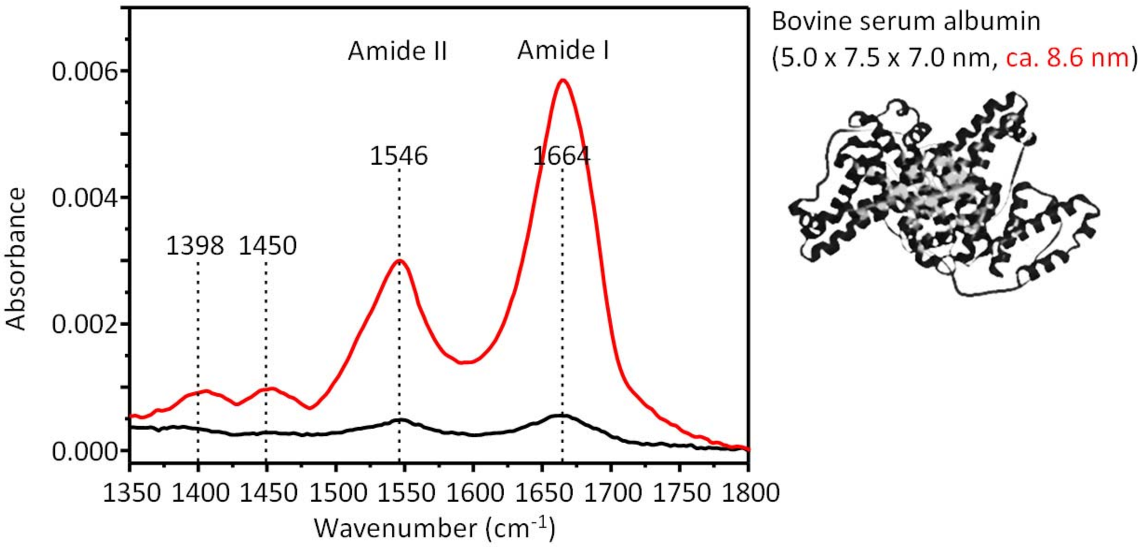

Supplementary Figure 14 | FT-IR absorbance spectrum of self-assemble monolayer of bovine serum albumin (SAM-BSA) on mesoporous Au film (Sample II). For comparison, FT-IR absorbance spectra of SAM-BSA on a flat Au surface without mesopores was also checked. No FT-IR signal is detectable from the BSA film on flat Au substrates.

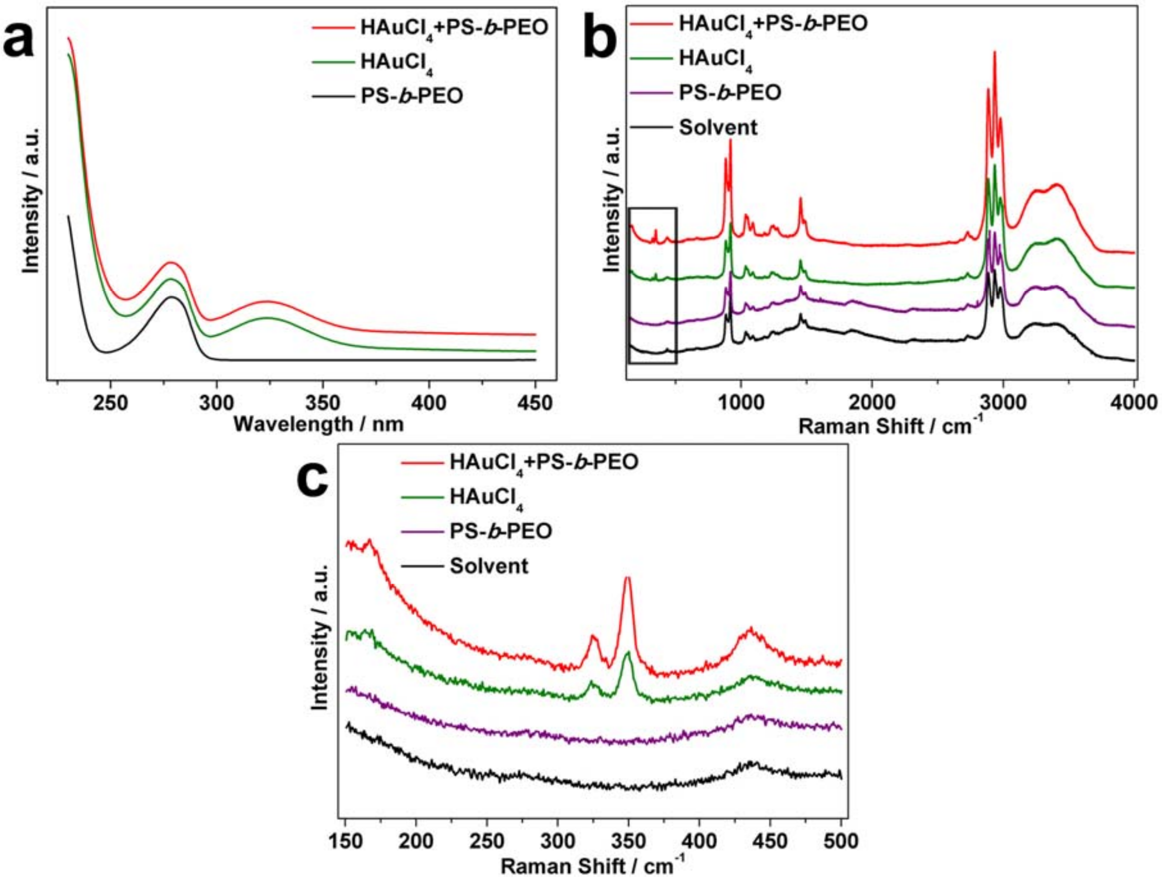

Supplementary Figure 15 | Investigation of coordination environment of the Au species dissolved in electrolyte. (a) UV-Vis and (b, c) Raman spectra of different solutions (Mixed solvent, and mixed solvent containing both PS-*b*-PEO micelles and $HAuCl_4$, only PS-*b*-PEO micelles, and only $HAuCl_4$, respectively.). (c) Enlarged rectangular region in panel (b).

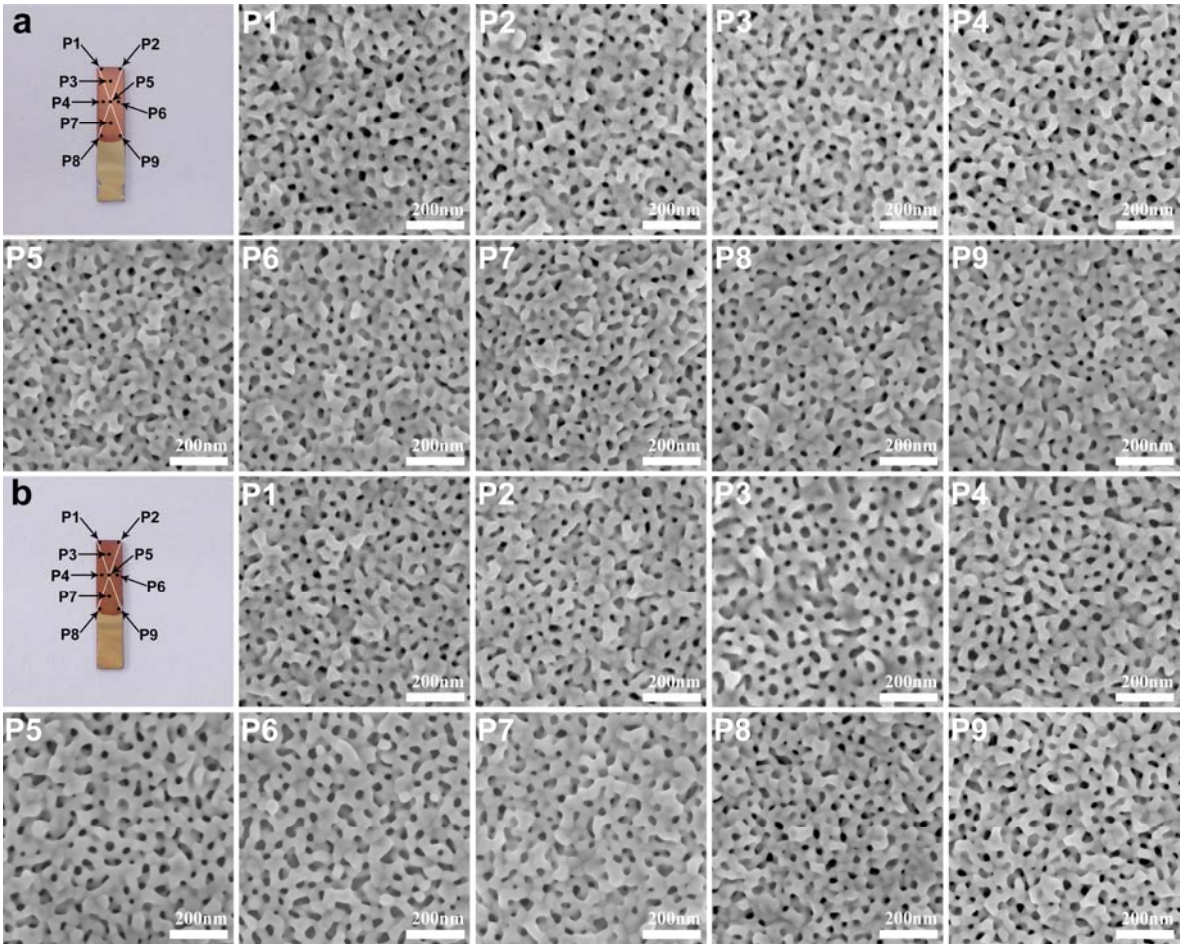

Supplementary Figure 16 | SEM images at various locations of mesoporous Au films prepared with a typical electrolyte containing PS_{18000}-*b*-PEO_{7500} micelles and 3 mL THF as solvent. (a) and (b) Two films are separately prepared from two different batches of electrolytes.

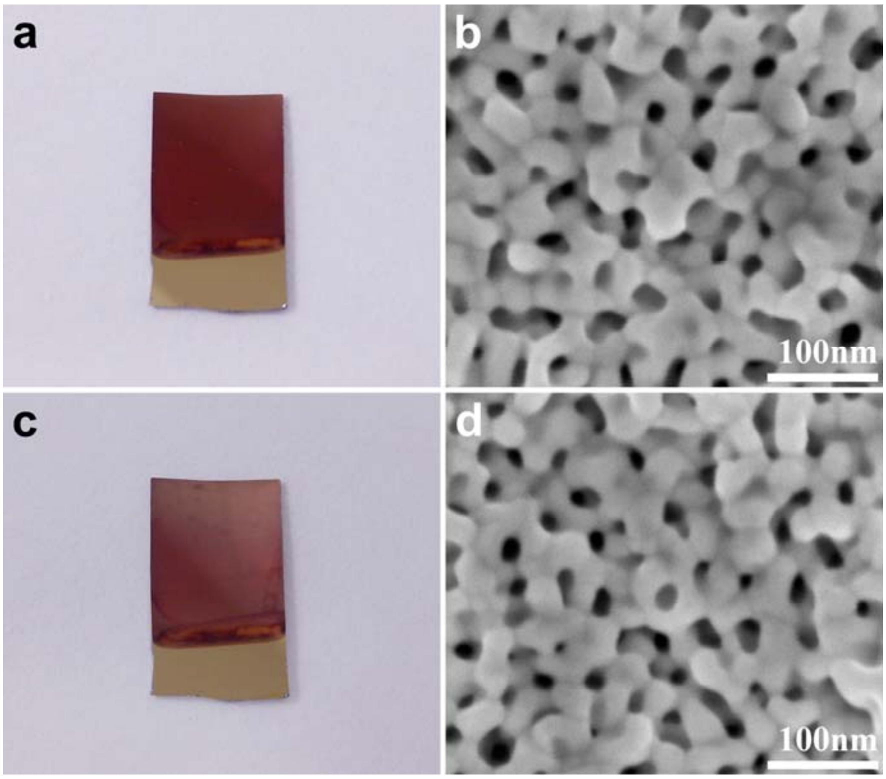

Supplementary Figure 17 | (a,c) Typical photographs of the mesoporous Au films (a) before and (c) after the peel-off test, and (b, d) typical SEM images of the mesoporous Au films (b) before and (d) after the peel-off test. The test samples (mesoporous Au films) are prepared with a typical electrolyte containing PS_{18000}-*b*-PEO_{7500} micelles and 3 mL THF as solvent.

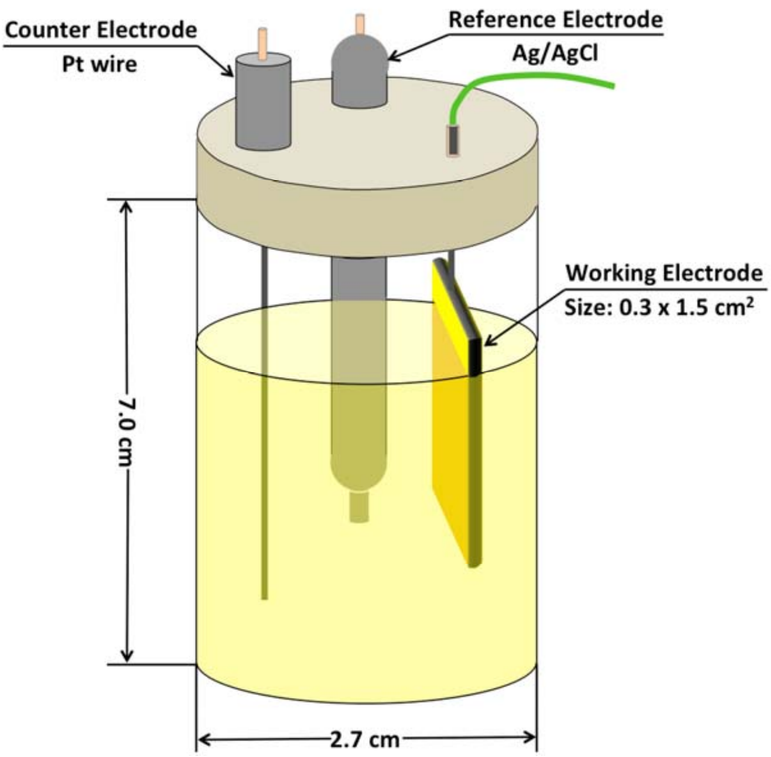

Supplementary Figure 18 | Schematic illustration of the used electrochemical cell for preparation of mesoporous Au films. The geometric cell size and the optimal size of working electrode are also noted.

Supplementary Note 1

Note for Supplementary Figure 1. At the early stage of Au deposition, the Au films do not grow uniformly on the substrates. So, it is hard to say the exact film thicknesses. But, if we assume that the Au film thicknesses are increased at constant growth rate (as shown in **Supplementary Figure 6**), the average film thicknesses are estimated to be (a) 1 nm, (b) 4 nm, and (c) 11 nm, respectively.

Supplementary Note 2

Note for Supplementary Figure 7. The estimation of ideal surface area can be calculated as follows. We assumed a monolayer of mesoporous Au film (prepared under the typical condition, Sample II). The average pore size and the average wall thickness were estimated to be 25 nm and 25 nm, respectively, from SEM observation. Assuming that the film volume is 125 nm (in length), 100 nm (in width), and 25 nm (in height), respectively, the film possesses 3 spherical mesopores and 4 hemi-spherical mesopores, as shown in the following image.

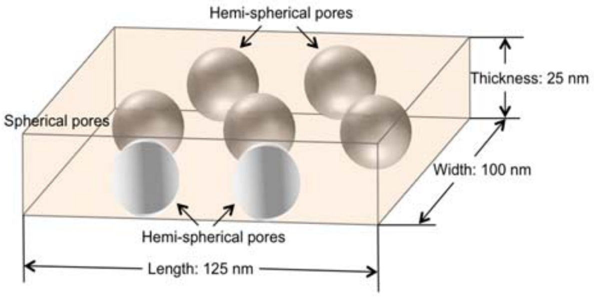

Supplementary Scheme: Schematic illustration of the monolayered mesoporsou Au film. The corresponding ideal length, width, and thickness of the film are noted.

By using this model, the total surface area (S) inside the mespores can be well obtained by considering 5 spherical mesopores.

$S = 5 \times 4 \cdot \pi \cdot R^2 = 5 \times 4 \times 3.14 \times (12.5 \times 10^{-9})^2 = 9.8125 \times 10^{-15}$ (m^2)

The bulk film volume of this model (V) can be calculated as follows.

$V = (125 \times 10^{-7}) \times (100 \times 10^{-7}) \times (25 \times 10^{-7}) = 3.125 \times 10^{-16}$ (cm^3)

Therefore, the volume-normalized surface area (P) can be obtained as follows.

$P = S \cdot V^{-1} = 31.4$ (m$^2 \cdot$cm^{-3})

This calculated value is a little less than the value obtained from the electrochemical measurement (49.1 m^2 cm^{-3}), because the actual pore surface in the obtained mesoporous Au films are bumpy (not smooth). High resolution TEM images also shows bumpy surface with atomic steps and kinks (**Fig. 2 & Supplementary Figure 8**), which increase the surface area rather than we expect.

Supplementary Note 3

Note for Supplementary Figure 16. Our approach shows a fairly high reproducibility (100 %). Actually, we repeated the same experimental procedure many times, and we obtained exactly the same mesoporous structures. Even at the edge parts, the same mesoporous structures were steadily formed. To illustrate the high reproducibility of our process, two films were separately prepared, and the SEM images obtained at different locations were displayed here.

Supplementary Note 4

Note for Supplementary Figure 17. The adherence between the electrodeposited mesoporous Au film and substrate was studied by a 90° peel-off test, which was conducted on the film sample using a 3M scotch tape. The tape was peeled-off from the mesoporous Au film surface by applying a fixed force of 5.0 ± 0.1 N with a test distance of 20 mm. After the peel-off test, it was observed that the deposited Au film is still adhered on the substrate. The samples before and after the peel-off test were observed by SEM, which further confirmed that the original mesoporous structures were well retained without deformation of the mesopores and formation of any cracks/scratches on the surface.

Supplementary Note 5

Note for Supplementary Figure 18. Beside the optimal conditions, the present method is versatile to large substrates and flexible conducting substrates. By using the optimal condition, a large-sized substrate of 3.00 cm^2 (1.5 cm × 2.0 cm) also can be used for the electrodeposition.
